# Artificial Intelligence in Critical Care: Promise, Peril, and the Path Forward

**DOI:** 10.7759/cureus.85323

**Published:** 2025-06-04

**Authors:** Amber Kumar, Akhil Taneja, Yogendra Pal Singh, Gaurav Pratap Singh, Saurabh Jain, Seema Meena

**Affiliations:** 1 Critical Care Medicine, Max Super Specialty Hospital, Patparganj, Delhi, IND; 2 Anesthesiology and Critical Care, Max Super Specialty Hospital, Patparganj, Delhi, IND

**Keywords:** artificial intelligence, clinical decision support, critical care, machine learning, predictive analytics

## Abstract

Artificial intelligence (AI) is poised to transform critical care by enhancing clinical decision-making, predictive accuracy, and operational efficiency. This editorial explores the expanding role of AI in intensive care units (ICUs), highlighting emerging applications such as predictive analytics, diagnostic imaging, real-time monitoring, and clinical decision support systems. Alongside these advances, the integration of AI presents critical challenges related to ethical implementation, regulatory oversight, clinician trust, and data interoperability. Drawing from current case studies and healthcare trends, this perspective underscores AI's potential to improve patient outcomes and optimize resource utilization. However, without rigorous validation, transparent practices, and a firm commitment to patient-centered design, AI risks becoming a liability rather than an asset. Sustainable adoption will require interdisciplinary collaboration, continuous evaluation, and the preservation of human connection at the heart of technologically supported care.

## Editorial

Integrating artificial intelligence (AI) into critical care: promise and prudence

As digital innovation reshapes healthcare, AI has emerged as a particularly promising tool in the high-stakes domain of critical care. With the healthcare system becoming increasingly digital, AI is notable in its ability to redefine clinical workflows and outcomes. AI is being increasingly viewed as the turning force in contemporary medicine, especially in the high-stakes environment of critical care. In the intensive care unit (ICU), where clinical success hinges on the quick interpretation of data and the timely making of decisions, AI is a paradigm that is not simply theoretical but extremely pertinent to modern practice [1]. However, in the context of increasing interest, there is one significant issue to be addressed: are these technologies ready for implementation at the bedside? Although the potential of AI is huge, its clinical suitability needs scrutiny with attention. Most of the healthcare professionals are still apprehensive, especially because of the black box-like nature of most AI algorithms, that is, their internal workings are often opaque, making it difficult to understand how specific decisions or predictions are generated. Furthermore, most of these models have not undergone prospective clinical validation to confirm their real-world applicability in intensive care settings [1].

Broadly speaking, as the ability of machines to imitate intelligent human action through computational algorithms, AI has come a long way in medicine. Its uses range from radiologic interpretation, predictive analytics, clinical decision support, simulation-based training, surgical robotics, and data-driven drug discovery [2]. Critical care stands apart, as ICU patients often depend on life-sustaining technologies such as mechanical ventilation and vasopressor support. These settings produce vast amounts of clinical data from monitors, lab reports, and imaging devices, presenting both a challenge and an opportunity for early, informed decision-making [3]. One of AI's most promising applications is predictive analytics. Clinical decline in the ICU can be sudden, and early warning systems based on machine learning (ML) are under development to identify subtle changes in physiological parameters that may herald events like sepsis or cardiac arrest. These systems combine historical clinical information with real-time input to improve early detection. Meanwhile, as ICU care becomes more complicated, cognitive burdens on clinicians have also increased, frequently postponing the identification of patient deterioration. New tools such as natural language processing and data mining are now applied to siphon out useful patterns from unstructured records. While AI-driven alert systems promise much, the vast majority are untested in potential future clinical trials despite optimistic retrospective performance [1].

AI at the crossroads of potential and clinical practice

AI holds significant promise in healthcare, offering the potential to enhance diagnostic accuracy, streamline workflows, and support clinical decision-making, especially in complex environments like critical care. AI is reshaping how diagnosis, prediction, and clinical decision-making are approached in modern medicine [2]. Over the past two decades, ML has emerged as a key driver of innovation, contributing to diagnostic tools, predictive models, and clinical decision support systems (CDSS). While some AI-based applications have received regulatory approval, widespread adoption remains limited. Barriers consist of issues regarding the transparency, consistency, dependability, and generalizability of algorithms, problems that are especially sharp in critical care [3]. In the ICU, most AI applications remain experimental and narrow in scope. Typical applications include predictive tools for early sepsis detection, using real-time clinical and laboratory data to identify high-risk patients potentially hours before overt clinical signs, and reducing clinician workload through automated documentation [1]. Alert systems based on AI are also being utilized to triage alarms, with the hope of minimizing alarm fatigue and having providers concentrate on high-risk patients. Though there are significant efficiency improvements provided by these technologies, their use should support clinical judgment and not supplant it. Predictive analytics is one of the most powerful applications of AI in critical care. These systems handle high amounts of structured and unstructured data, from demographics and comorbidities to lab results, imaging, and treatment history, to detect early warning signs of decline. Such data in numerical or text form serves as the basis for sophisticated prediction models aimed at informing point-of-care choices [3].

Diagnostic imaging, too, has revolutionized with the help of ML and deep learning (DL) technologies. They are particularly suited to analyze intricate imaging modalities like computed tomography (CT), magnetic resonance imaging (MRI), and X-rays. In pulmonary diagnostics, AI has been utilized for improving image quality as well as for analyzing thoracic dynamics and lung nodules [4]. CDSS platforms have themselves become more dynamic with ML and DL capabilities that allow them to learn and respond to changing patient states [5]. Such adaptability is a must in critical care, where physiological parameters can shift within minutes. Clinical adoption remains guarded despite these developments. Many clinicians feel uncomfortable with algorithmic recommendations they do not fully understand. To become accepted, CDSS will need to show clinical usefulness, as well as interpretability, workflow integration, and decision support that augments, not replaces, clinician judgment. ICUs produce an enormous amount of high-resolution data from bedside monitors, lab systems, and electronic health records [1]. Though this data is invaluable to care, it burdens clinicians and derails decision-making. Protocols try to normalize responses, but cannot account for individual patient variation [4]. Recent supervised learning approaches can find clinically important patterns without human designers defining rule sets [6]. Such systems can pick up early warning signs of clinical deterioration-decompensation, sepsis, or organ dysfunction by analyzing multimodal and time-series information. As compared to the old-school, manually designed-feature dependence methods, these data-intensive systems learn from real-world data directly, enhancing scalability as well as minimizing bias. While performance does need extensive validation across heterogeneous patient populations to prevent misclassification or alert fatigue, its dependence on local workflows and data structures continues to be a major hurdle.

Remote patient monitoring has become more popular, especially during infectious epidemics, when reducing clinician exposure is important. Current systems commonly use audiovisual feeds to achieve situational awareness at a distance, but usually do not integrate key physiological measurements like oxygen saturation, respiratory rate, or blood pressure [7]. Functional restrictions, coupled with expense, technological sophistication, and lack of interoperability, limit wider application. Also, there are growing ethical concerns, particularly with networked devices that can potentially expose patient information through insecure platforms or social media. There is a need to provide strong privacy protection and regulation [8]. Next-generation monitoring technologies need to incorporate real-time physiological inputs, provide clinically actionable information, and be built with cybersecurity as an inherent component. Most current real-time CDSS applications still use static, rule-based systems that are not flexible enough for dynamic care settings [6]. AI algorithms, if well-trained on large and diverse datasets, have the capability of beating such conventional systems by providing individualized, multi-parameter predictions [1]. However, all this is still met with resistance from clinicians by way of objections over trust, accountability, and alert reliance. For intricate diagnostic cases, human judgment is indispensable. AI algorithms also rely on properly structured input data, such as labs, vitals, and imaging, whose ranges can have a significant influence on model output. To succeed, real-time AI applications need to optimize sensitivity and specificity, reducing false negatives and false positives alike. Their worth ultimately resides in being able to natively integrate into clinical workflows, without trading away transparency, interpretability, or patient safety.

Engineering intelligence: training, ethics, and integration of AI in critical care

ML and other types of computer intelligence have emerged as potent analytic tools across the different biomedical fields [3]. In critical care, an interdisciplinary data-intensive field, ML is especially advantageous. Such technologies are already being deployed across a broad array of challenges, from hospital epidemiology through neurocritical care to real-time modeling of ventilation, hemodynamic variables, and metabolic profiles [9]. This scope of application signifies that AI can not only handle immense amounts of ICU data but also inform individualized, adaptive treatment approaches. AI processes apply a range of learning methods, such as supervised, unsupervised, and reinforcement learning. These techniques allow AI to carry out activities such as signal interpretation, outcome prediction, surveillance, and clinical decision support. Notably, ICUs were among the first adopters of computerized patient data systems, providing a solid groundwork for embedding AI technologies into everyday clinical practices [1].

Nevertheless, the success of AI is greatly dependent on the quality and comprehensiveness of its training and validation procedures. Before their release into clinical settings, extensive preprocessing in the form of feature cleaning, normalization, and statistical modeling is required. Sensor- or electronic health record-based data needs to be carefully prepared. Model construction also calls for systematic testing, hyperparameter optimization, and architectural improvement. While there have been swift developments in algorithm construction, the practical application of AI continues to rely on whether or not it can handle heterogeneous, high-quality data that captures clinical heterogeneity [3]. Ethical as well as practical issues have also to be resolved alongside technical innovation. AI algorithms are many times opaque, and clinicians remain reluctant to trust tools they are not able to fully understand [10]. This problem is particularly critical in critical care, where choices need to be made urgently and decisively. Live learning systems that develop beyond their initial training parameters could generate unforeseen outputs, and this raises very critical concerns. Hence, ethical deployment also has to incorporate technical correctness and well-defined accountability for results.

Access to patient data also presents difficulties. Developers may need access to real-world data to enhance model performance. But providing sensitive information with insufficient protection can undermine both patient confidentiality and institutional trustworthiness [11]. To this end, an aggregated ethical framework would be developed through collaboration between clinicians, developers, and ethicists. The framework would encourage innovation while preserving public trust [11]. Even if institutions are provided with high-quality data, most don't have the infrastructure in place to process it efficiently. For instance, the 24- to 48-hour preclinical deterioration window is dense with predictive information. However, few ICUs possess the capability of capturing, storing, and crunching this high-resolution data within a reasonable period [12]. Proprietary data systems, especially those that archive signals such as electrocardiograms or oxygen saturation, usually don't support interoperability. These isolated platforms are a hindrance to both model development and bedside utility. Moreover, most hospitals still maintain legacy software systems with restricted capabilities, so the representation of timely data is fragmented or not understood. Such limitations directly impinge on the potential for AI in assisting clinical decision-making at the bedside [13].

Beyond the algorithm: human-centric innovation and the future of AI in the ICU

ML-based platforms have proved to possess outstanding abilities in identifying patterns within huge, intricate datasets. Natural language processing, signal understanding, and picture recognition are some of the applications that are now commonly used for diagnostics and clinical categorization [3]. Among them, DL has proved especially promising to improve diagnostic precision and decrease error rates. The outcome, though, is highly subject to the access to good-quality, secure training datasets [11]. AI systems usually take information from three main sources: point-of-care monitoring devices, public health databases, and real-time feedback systems. ICUs, for example, produce large amounts of data from automated monitors every day. Public registries, however, provide population-level modeling and larger epidemiological understanding. As AI technologies become more integrated into clinical settings and are increasingly treated as high-risk medical devices, they must adhere to strict standards for transparency, human oversight, and performance validation [10,11].

Despite accelerating progress, algorithmic bias remains a pressing issue. If AI models are trained using datasets that mirror past inequalities or are not demographically diverse, they will reinforce or even exacerbate disparities in the delivery of care [13]. Diagnostic algorithms rooted in such unbalanced information might perform suboptimally among underrepresented groups. Resolving this calls for explicit incorporation of diversified datasets and ongoing fairness monitoring through the model's development and clinical deployment. To reach their maximum potential, AI instruments need to be properly integrated into clinical infrastructure. ICUs already record thousands of pieces of data per patient, from laboratory data and diagnostic codes to medication lists and nursing evaluations. Although most institutions have centralized databases to enable AI analysis, real-world implementation relies on being able to interface seamlessly with electronic health record systems. If outputs are not intuitive, timely, and easily interpretable by clinicians, AI risks being underutilized or ignored.

Clinician buy-in is critical to effective AI implementation. Research shows that systems created with direct input from physicians and nursing staff have a greater likelihood of fitting into established workflows and being accepted within the organization [14]. End-user co-design results in more intuitive and trustworthy tools. Human supervision, contextual awareness, and empathetic care are still at the core of safe and ethical healthcare provision. Ahead, AI innovation in healthcare remains on a speedy trajectory. Between 2015 and 2019, more than 20 health-technology startups were established in Japan alone, indicating international interest in AI-based innovation [2]. In intensive care, AI use continues to spread across areas like disease identification, patient outcome prediction, and treatment assistance in fields like mechanical ventilation, sedation management, renal replacement therapy, and fluid resuscitation [15]. DL models have also been proven to predict life-threatening events, including cardiac arrest and acute respiratory failure, sometimes even before clinical symptoms manifest themselves [16].

This technological development accompanies a larger movement in healthcare, away from generalized public health interventions toward personalized, precision-medicine care. AI stands to facilitate this shift by allowing time-series analysis, anomaly detection, and personalized clinical optimization [17]. But across-the-board use of AI also has challenging ethical and social consequences. Equitable access, patient data protection, and sensitivity to socioeconomic inequalities are among the issues that must be actively managed [11]. Notably, AI must not undermine the human perception of care. In intensive care environments such as ICUs, patients are likely to feel isolated or neglected if automation replaces person-to-person contact. Issues of depersonalization, alarm overload, and reduced emotional support are real and need resolution. The nurse-patient relationship, sometimes the foundation of recuperation in intensive care, must be maintained and safeguarded [15,17]. In total, AI has the potential to transform critical care, but only by careful deployment. Clinical acumen, moral responsibility, and a strong desire to protect the human aspects of medicine will be necessary to ensure these technologies reinforce, not erode, patient-centered care.

Real-world integration of AI in critical care: case studies, stakeholder perspectives, and the road ahead

One of the most intensively researched areas of AI application in intensive care addresses the prediction of clinical deterioration. DL models have been pre-trained on multivariate time-series data to predict treatment requirements and identify physiological trends that might herald negative outcomes. The tools are designed not only to predict risk but also to enable timely clinical intervention. Japan has spearheaded several high-profile real-world initiatives in this area. Keio University's Aesthetic Robotics group, together with Tokyo Medical and Dental University, Waseda University, and Tokyo Denki University, is working on an AI-based monitoring system that employs computer vision to estimate vital signs of patients. The system, which is tailored for application in emergency transfers in disasters, is under simulation-based testing [1]. Another project, spearheaded by Keio University and the University of Tokyo, merges AI with virtual reality to advance patient safety training. Initial findings indicate that AI-powered virtual agents enhance critical care training performance over conventional methods. Similar initiatives have been reported in other countries as well, for example, in the United Kingdom, with AI increasingly being integrated into medical education and simulation-based learning frameworks to enhance clinical reasoning and preparedness [1,16]. Also in development are time-series modeling software, such as Trace, to identify early risks of sepsis within a 10-hour window of prediction [2].

Many feel uneasy at the possibility of AI replacing human clinicians, particularly where face-to-face contact is lost [17]. Concerns also encompass alert fatigue, system obscurity, and data surveillance overload. To manage these challenges, ethical AI adoption should prioritize transparency, accountability, and fairness, particularly where patients have little to no control over the utilization of their data [16]. Regulatorially, initiatives for governance and operational clarity are ongoing. In 2021, the World Health Organization provided guidelines for AI ethics and governance in healthcare, with an emphasis on clearly articulated standards [14]. In a similar vein, the United States Food and Drug Administration has established policies for AI-based medical devices that include algorithm lifecycle management, post-market monitoring, and the need for human intervention protection measures [15]. For AI to be effective in ICUs, it needs to be applied within an infrastructure that allows for quick communication and data-driven decision-making. Such environments require real-time processing of intricate physiological data. Applications currently encompass detection of disease, scoring of severity, management of ventilators, and recruitment for clinical trials [2]. Research has found that AI-based systems tend to outperform traditional risk score instruments in forecasting unfavorable outcomes like cardiac arrest and respiratory failure, consistently reporting higher area under the curve (AUC) measurements [1,2].

However, the ongoing impediment to implementation is the lack of alignment between developers and frontline clinicians. Numerous AI solutions are developed with little clinical involvement, resulting in systems that are challenging to decipher or mismatched with workflow requirements [18]. This is part of what is commonly referred to as the "AI deployment bottleneck," wherein promising models fail to scale from lab to practice. Compounding this is the infrastructural limitation of antiquated systems and limited capacity for integration. It takes early and sustained cooperation between developers, healthcare workers, and institutional leaders to bridge these gaps [14,19]. Educating clinicians in AI basics is also crucial in establishing trust and deploying safely. Appropriate guidelines and mechanisms for ethical review can be used to guarantee that AI outputs are transparent, trustworthy, and clinically actionable [2]. National ethics commissions and regulatory authorities play a major role in monitoring the ethical use of these tools. Future policy might also have to include burgeoning disciplines like cybernetics and human-machine interaction, especially imaging and diagnostic applications [2]. Last but not least, the future of AI in medicine will fundamentally transform medical education. As these technologies become routine in clinical practice, physicians of the future need to be ready to engage with them competently and confidently. Curricula should cover digital literacy, algorithmic critique, and education in collaborative human-AI decision-making. Honing these skills is necessary to make sure that AI strengthens instead of degrading the quality, safety, and humanity of patient care [20,21].

Conclusion

With continued advancements in algorithmic design and clinical integration, AI holds increasing potential to improve outcomes in intensive care. Most ICU-specific models, however, are still in development stages and lack the prospective validation required for safe deployment into clinical practice. Ongoing challenges such as data heterogeneity, system integration constraints, regulatory ambiguity, and decreased interpretability continue to hinder universal adoption. Despite such challenges, AI has the potential to augment early clinical deterioration detection, reinforce decision-making, streamline workflows, and enhance early detection and response to clinical deterioration. Leveraging its potential will take a collaborative approach based on ethical norms, clinical trust, and interprofessional collaboration. Patient and family engagement in design and implementation is also essential to maintain transparency as well as ensure that care remains person-focused. In the future, the research should focus on clinical validation, designing natural human-AI interfaces, and large-scale training of healthcare providers. Through careful, ethically informed, patient-centric strategies, AI has the potential to become an effective and safe assistant in delivering critical care.
